# Trade-Off Between Water-Absorption Reduction and Strength-Gap Recovery at Three KH560 Treatment-Solution Concentrations for Airport-Pavement Recycled-Aggregate Concrete

**DOI:** 10.3390/ma19184006

**Published:** 2026-09-20

**Authors:** Lihao Liu, Lei Xi, Mengming Li, Gaoyun Song

**Affiliations:** 1 Key Laboratory of Intelligent Health Perception and Ecological Restoration of Rivers and Lakes, Ministry of Education, Hubei University of Technology, Wuhan 430068, China; 18337727999@163.com (L.L.);; 2 Hubei Key Laboratory of Environmental Geotechnology and Ecological Remediation for Lake & River, Hubei University of Technology, Wuhan 430068, China; 3 School of Civil Engineering and Architecture, Hubei University of Technology, Wuhan 430068, China

**Keywords:** airport pavement recycling, recycled coarse aggregate, KH560 silane, water absorption, strength-gap recovery, nominal treatment-solution concentration

## Abstract

**Highlights:**

- KH560 treatment was assessed for one airport-pavement RCA at full replacement.
- Nominal solution concentration was distinguished from actual KH560 uptake.
- SS10 gave the highest 28-day means among the three treated RCA mixtures.
- SS12 showed 64.3% lower water absorption than untreated RCA, the largest observed difference.
- Water absorption and concrete strength favored different treatment levels.

**Abstract:**

This study investigated whether the nominal 3-glycidoxypropyltrimethoxysilane (KH560) treatment-solution concentration associated with the lowest recycled coarse aggregate (RCA) water absorption also yielded the greatest recovery of concrete strength. RCA obtained from a decommissioned airport pavement was treated using solutions with nominal KH560 concentrations of 8%, 10%, and 12% and used as 100% of the coarse aggregate. All mixtures had the same target grading and nominal effective water-to-cement ratio of 0.360. Relative to untreated RCA, the 10% group (SS10) showed 18.7% lower water absorption and a 22.7% lower crushing index. Its 28-day compressive, flexural, and splitting tensile strength means were 13.8%, 31.3%, and 26.4% higher than the corresponding untreated recycled-aggregate concrete means. The SS10 flexural-strength mean reached 94.7% of the natural-aggregate concrete mean. The 12% group (SS12) showed the lowest water absorption at 64.3% below that of untreated RCA, but lower strength means than SS10. Thus, the concentration favored by water absorption differed from that favored by the three measured strength properties. The results provide a preliminary laboratory basis for selecting KH560 treatment levels for further evaluation of the investigated airport-pavement RCA.

## 1. Introduction

Recycling concrete from decommissioned pavements can conserve virgin mineral resources and reduce the volume of material requiring off-site disposal. The structural value of this route, however, depends on whether the recovered aggregate can support the mechanical demands of a new pavement. Recycled coarse aggregate (RCA) retains adhered mortar, crushing-induced defects, and additional interfacial zones. These features increase water absorption, complicate mixture–water control, and can weaken stress transfer in recycled-aggregate concrete. The consequences are particularly relevant to rigid pavements, for which compressive resistance supports load distribution while flexural and tensile resistance govern slab cracking and serviceability [1,2,3,4,5,6].

Concrete from a defined airport-pavement renewal project offers a traceable demolition source, although traceability does not establish uniform parent-concrete quality. Recycling this material may retain mineral resources within successive infrastructure cycles. Source characterization, impurity removal, grading, moisture control, and pavement-specific performance assessment remain necessary before structural reuse [7,8,9].

RCA modification has therefore developed from simple contaminant removal toward treatments that alter the residual mortar and aggregate surface. Reviews commonly group these routes into mechanical removal, densification, impregnation, coating, carbonation, and combined treatments. Their performance depends on the original concrete, treatment intensity, reagent distribution, and the target application [10,11]. Recent work with pretreated 100% recycled coarse aggregate confirms that aggregate upgrading can reduce water absorption and crushing index while improving concrete performance. It also shows that the final response remains sensitive to material design and treatment conditions [12]. A useful treatment must therefore be evaluated at both the aggregate and concrete scales rather than selected from one isolated property.

Silane-based treatments, including the epoxy-functional coupling agent 3-glycidoxypropyltrimethoxysilane (KH560), are relevant because they can modify surface wettability and restrict accessible water pathways without requiring removal of all adhered mortar. Previous studies, however, used materially different treatment scales and design objectives. Surface or integral silane treatment of recycled-aggregate concrete improved transport-related durability, although integral addition could reduce compressive strength [13]. Impregnation of coarse RCA with 8 wt% silane reduced water uptake and chloride transport, but produced a slight compressive-strength decrease [14]. A 10% treatment improved the compressive response of recycled brick aggregate in mixed-aggregate concrete [15]. Other KH560 studies combined the coupling agent with fibers, making it difficult to isolate concentration as the controlling variable [16]. A 2024 orthogonal study also used 8%, 10%, and 12% KH560, but it simultaneously varied treatment time and RCA content (20-60%). Because the outcomes were water absorption, slump, and 28-day compressive strength, concentration was not isolated from the other factors [17]. Ethylene–vinyl acetate copolymer (EVA)/KH560 co-modification likewise examined a polymer-assisted system rather than a standalone KH560 treatment [18]. Collectively, these studies demonstrate treatment potential but do not resolve how KH560 concentration redistributes water absorption, crushing resistance, and multiple concrete-strength responses under full RCA replacement.

Recent studies, discussed in Materials, further define the controls required for such an assessment. Polymer impregnation and coating are recognized as viable RCA-modification routes, but compatibility and strengthening efficiency remain process-dependent [19]. RCA water-absorption measurement also directly affects absorption correction and the calculated effective water-to-binder ratio [20]. Combined cement-slurry and methyl-sodium-silicate treatment can improve water absorption, crushing resistance, and concrete performance, yet the composite process does not isolate the concentration response of a single silane coupling agent [21]. These findings support an experimental design that holds grading constant, uses a common nominal effective mixture-water basis, and evaluates water control and strength recovery together.

The unresolved question is whether the nominal treatment-solution concentration that minimizes RCA water absorption also maximizes concrete strength-gap recovery. Here, strength-gap recovery denotes the fraction of the untreated-RAC-to-NAC strength deficit recovered by treatment. NAC is natural-aggregate concrete, and RAC is concrete containing untreated RCA. This metric differs from strength retention relative to NAC and the percentage strength increase relative to RAC. The definitions and reference strengths are specified in Section 2.5.

This study addresses that question using one airport-pavement RCA source at 100% coarse-aggregate replacement and three nominal KH560 treatment-solution concentrations. The target grading and nominal effective water-to-cement ratio were common to all mixtures. By comparing aggregate absorption, crushing index, and three 28-day concrete strengths within the same program, this study determines whether the two levels of performance favor the same treatment. The three tested concentrations provide a preliminary laboratory comparison rather than a continuous concentration-response model.

## 2. Materials and Methods

### 2.1. Raw Materials and RCA Source

RCA was recovered from decommissioned cement-concrete pavement slabs in the airfield area of a civil airport in northwestern China. Project documentation identified the material as originating from a single pavement-renewal project. The pavement age, original strength grade, carbonation depth, and chloride condition were not documented. The concrete constituents comprised P·C 42.5 cement supplied by Hunan Xiangxiang Chengmei Cement Co., Ltd. (Xiangtan, Hunan, China), natural sand, continuously graded natural coarse aggregate (NCA), RCA, and water. A WH-H (I) polycarboxylate water-reducing admixture had reported water-reduction and bleeding ratios of 28% and 11%, respectively. KH560 (3-glycidoxypropyltrimethoxysilane; CAS No. 2530-83-8) was supplied by Dongguan Kangjin New Materials Technology Co., Ltd. (Dongguan, Guangdong, China) Anhydrous ethanol and deionized water were used to prepare its treatment solutions. The available product documentation identified the cement as P·C 42.5 but did not report batch-specific chemical composition or Blaine specific surface area.

Table 1 summarizes the baseline aggregate properties. Untreated RCA had higher water absorption and crushing index than NCA.

### 2.2. Wet Processing and KH560 Treatment

The processing sequence was designed to retain the target coarse fraction while removing visible contaminants and weak surface material. Steel, wood, asphalt fragments, and other identifiable impurities were removed before staged jaw crushing and screening. The coarse material was then washed with an 8 MPa water jet at a working distance of 250–300 mm. Each 15–20 kg batch was washed for 5 min, with the aggregate layer maintained below 80 mm and turned two or three times to improve exposure. Washing was stopped when the discharged water was substantially clear and no visible loose fines or contaminants remained on the aggregate surface.

**Figure 1:**
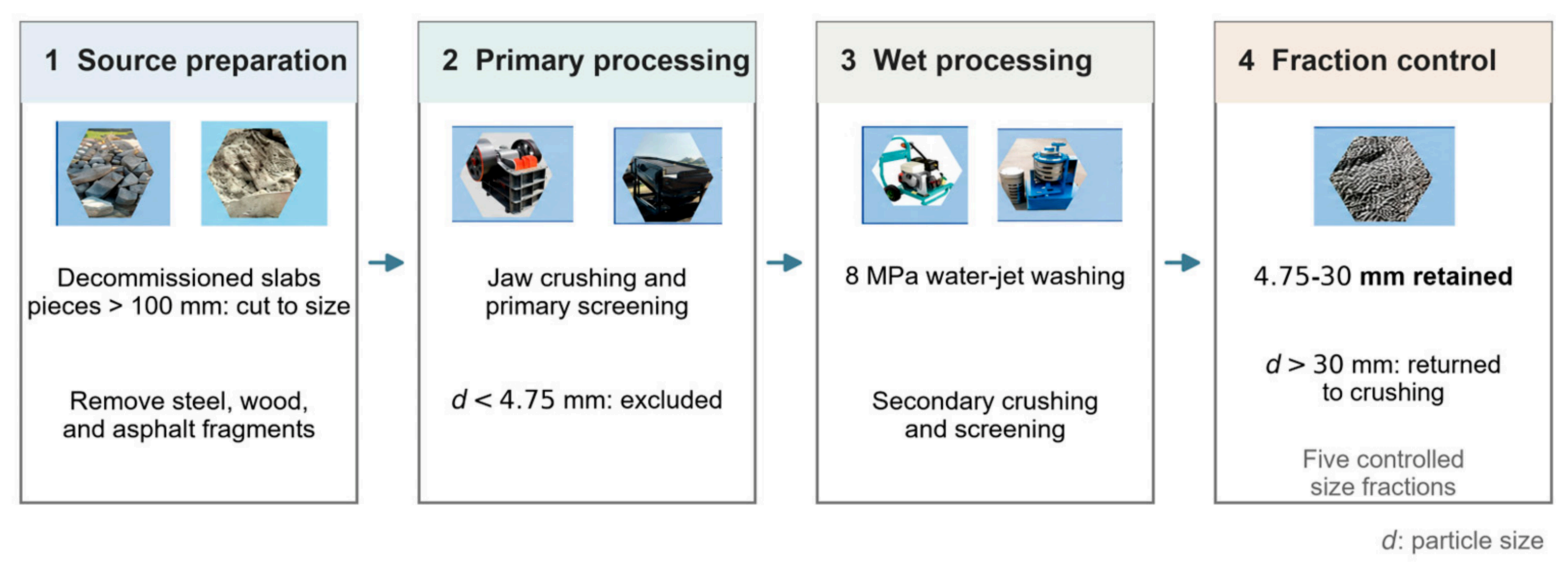
Wet processing of recycled coarse aggregate from airport pavement concrete.

After washing, the material was allowed to drain before secondary crushing and screening. Particles smaller than 4.75 mm were excluded from the coarse-aggregate stream, whereas particles larger than 30 mm were returned to crushing. The retained 4.75–30 mm fractions formed the wet-processed RCA used in the treatment program. This sequence combined source sorting, controlled size reduction, high-pressure washing, and closed-loop recovery of oversized particles (Figure 1).

Nominal KH560 treatment-solution concentration was defined as KH560 mass divided by total treatment-solution mass, expressed as a percentage. SS8, SS10, and SS12 denote the 8%, 10%, and 12% formulations, respectively. The ethanol-to-deionized-water mass ratio was fixed at 8:1. These labels describe solution composition and do not denote KH560 uptake per unit aggregate mass.

On a 100-unit solution-mass basis, the KH560:ethanol:water formulations were 8:81.78:10.22 for SS8, 10:80:10 for SS10, and 12:78.22:9.78 for SS12. The 10% formulation therefore corresponded to a 1:8:1 mass ratio. Each solution was mixed to visual homogeneity and allowed to stand for 3 min for pre-hydrolysis. The same preparation sequence was applied to all three concentrations.

Actual KH560 uptake by RCA was not measured; therefore, the stated concentrations describe treatment-solution composition rather than absorbed reagent dose.

**Figure 2:**
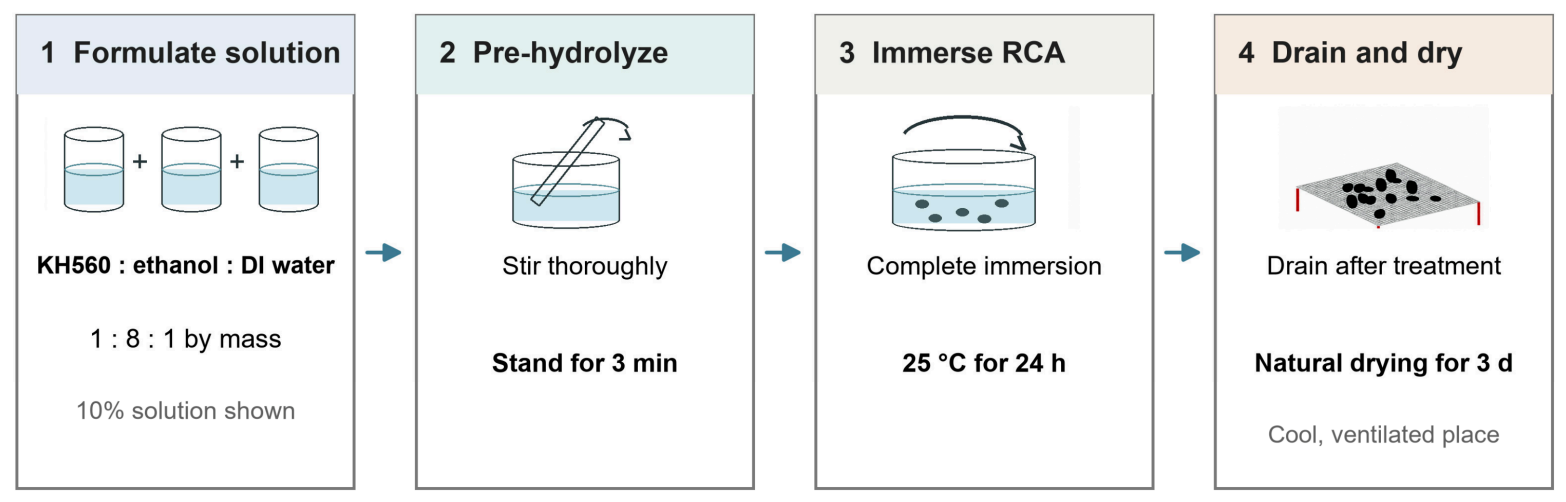
KH560 treatment of recycled coarse aggregate.

Air-dried RCA was fully immersed in its designated solution for 24 h at 25 °C, with intermittent stirring. After draining, it was naturally dried for 3 d in a cool, ventilated environment without high-temperature oven drying. The same immersion and drying conditions were used for all three formulations (Figure 2). This controlled procedure supported comparison among the three formulations.

The untreated RCA reference did not undergo ethanol-water immersion. Accordingly, comparisons of treated groups with RAC reflect the complete immersion-and-drying treatment, whereas comparisons among SS8, SS10, and SS12 evaluate nominal treatment-solution-concentration differences under the same procedure.

### 2.3. Controlled Grading, Mixture Design, and Research Program

**Figure 3:**
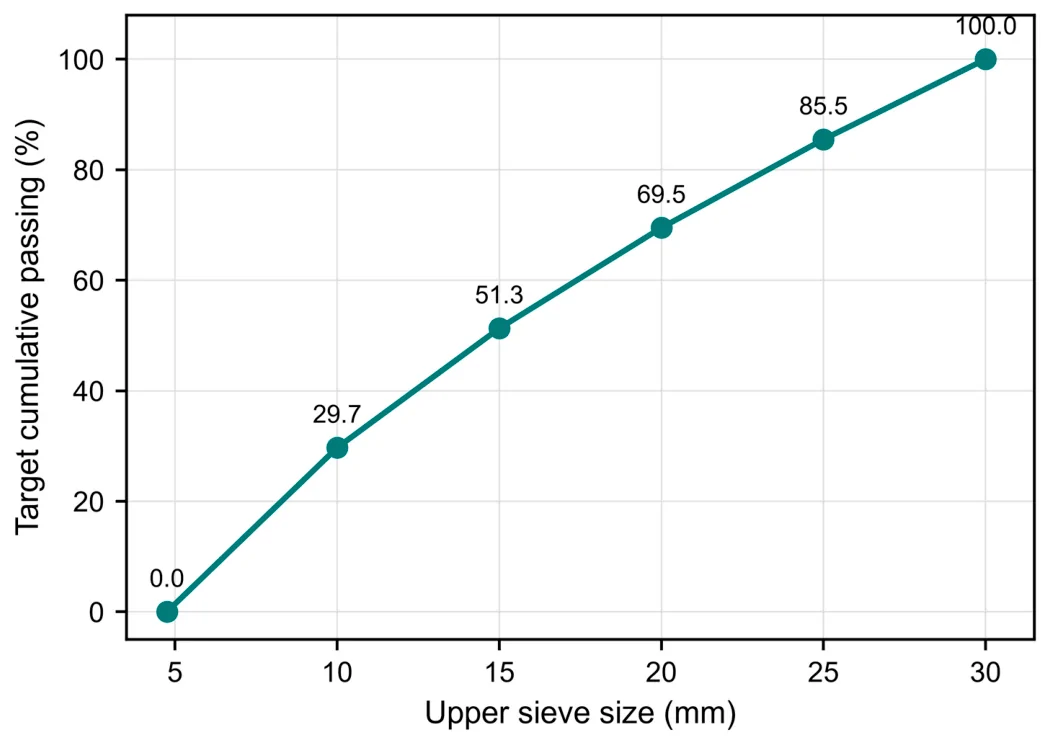
Target grading of the recombined coarse aggregate.

Coarse aggregate was separated into 4.75–10, 10–15, 15–20, 20–25, and 25–30 mm fractions and recombined using the reported fractal-grading approach [22,23,24]. Their mass proportions were 29.7%, 21.6%, 18.2%, 16.0%, and 14.5%, respectively. The corresponding target cumulative passing values were 0%, 29.7%, 51.3%, 69.5%, 85.5%, and 100% at sieve sizes of 4.75, 10, 15, 20, 25, and 30 mm (Figure 3).

The same target recombination was specified for NCA, untreated RCA, and all treated groups. This reduced planned grading differences between mixtures.

Five concrete mixtures were prepared. NAC contained NCA and served as the natural-aggregate reference. RAC contained untreated wet-processed RCA. SS8-RAC, SS10-RAC, and SS12-RAC contained RCA treated with the corresponding KH560 solution. Coarse-aggregate replacement was 100% in every RCA mixture, providing a stringent assessment of treatment effectiveness without dilution by natural coarse aggregate.

Cement, sand, total coarse aggregate, nominal effective water, and water-reducer contents were 570, 613, 1012, 205, and 2.85 kg m^−3^, respectively (Table 2). JGJ 55-2011 [25] provided the general mixture-design reference. The nominal effective water-to-cement ratio was 0.360. Absorption-compensation water was added separately and excluded from the 205 kg m^−3^ effective-water allocation. The calculation used the dry-mass basis in the mixture design.

Absorption-compensation water was calculated from RCA mass and the difference between RCA and NCA water absorption using Equation (1). Table 2 reports the resulting additional-water amounts. Table 3 summarizes the tests and the available specimen information.

The absorption correction was intended to reduce mixture-water differences. It specifies a nominal design basis rather than a measured free-water ratio for each batch.

(1) $$W_{\mathrm{add}}=m_{\mathrm{RCA}}\times \frac{A_{\mathrm{RCA}}-A_{\mathrm{NCA}}}{100}$$

where *W*_add_ is absorption-compensation water (kg m^−3^), *m*_RCA_ is RCA dry mass (kg m^−3^), and *A*_RCA_ and *A*_NCA_ are water absorptions (%). JGJ 55-2011 [25] provided the general mixture-design reference; Equation (1) states the compensation calculation used in this study.

### 2.4. Specimen Preparation and Testing

Aggregate properties were measured with reference to GB/T 14685-2022 [26]. The concrete program focused on comparing the 28-day compressive, flexural, and splitting tensile strengths of the five mixtures. It used 100 mm cubes for compression and splitting and 100 mm × 100 mm × 400 mm prisms for flexure, with GB/T 50081-2019 [27] as the method reference. A 150 mm × 300 mm cylinder configuration would be applicable to static elastic-modulus testing; however, static elastic modulus was not included among the properties investigated in this study.

All mixtures were tested using the same specimen geometry for each strength property. The available records do not establish whether specimen-size conversion factors were applied; equivalence to results obtained with other specimen geometries is therefore not claimed. The reported combination of 100 mm splitting specimens and a maximum aggregate size of 30 mm exceeds the 19 mm aggregate-size limit for this specimen cross-section specified for splitting tensile tests in GB/T 50081-2019 [27]. The splitting results are therefore treated as within-program observations rather than standard-compliant strength values.

Specimens were demolded after 24 h and cured to 28 d at (20 ± 2) °C and at least 95% relative humidity.

Each group comprised three independent aggregate samples or three concrete specimens from one batch, as applicable. Results are reported as mean ± sample standard deviation (SD), with the coefficient of variation (COV) calculated as $\mathrm{COV}=\frac{\mathrm{SD}}{\bar{x}}\times 100\%$. For each strength property, exploratory specimen-level comparisons among the five mixtures used one-way analysis of variance (ANOVA) followed by Tukey’s honestly significant difference (HSD) test at α = 0.05. These tests assume independent, normally distributed errors with equal variances; these assumptions were not established by the small dataset. The statistical results were verified using Python 3.12.10 and SciPy 1.18.0. Only one concrete batch was prepared per mixture, so between-batch variation cannot be estimated and mixture effects cannot be separated from batch effects. The p-values therefore describe exploratory comparisons within this experimental program, not independently replicated mixture-level effects.

### 2.5. Performance Evaluation Indices

For strength property *p*, let *f*_T,*p*_, *f*_RAC,*p*_, and *f*_NAC,*p*_ denote the reported group means for a treated mixture, untreated RAC, and NAC. Strength retention is $S_{p}=\frac{100f_{T,p}}{f_{\mathrm{NAC},p}}$. Relative strength increase is $I_{p}=\frac{100\left(f_{T,p}-f_{\mathrm{RAC},p}\right)}{f_{\mathrm{RAC},p}}$. Strength-gap recovery is $R_{p}=\frac{100\left(f_{T,p}-f_{\mathrm{RAC},p}\right)}{f_{\mathrm{NAC},p}-f_{\mathrm{RAC},p}}$. Thus, *R_p_* = 0% represents the RAC mean and *R_p_* = 100% represents the NAC mean. All three NAC−RAC denominators are positive. The index expresses the recovered reference strength deficit; it does not imply a physical repair mechanism.

Water-absorption reduction is $B=\frac{100\left(A_{\mathrm{RCA}}-A_{T}\right)}{A_{\mathrm{RCA}}}$, where *A*_T_ is the treated-aggregate absorption. Mean mechanical gap recovery is the arithmetic mean of the three property-specific *R_p_* values, with equal weights. A four-objective comparison uses *B* and the three separate *R_p_* values. A formulation dominates another only if it is no worse in every objective and better in at least one. These calculations summarize the group means.

## 3. Results

### 3.1. Aggregate Performance

NCA is an external reference; reductions are calculated relative to untreated RCA.

**Figure 4:**
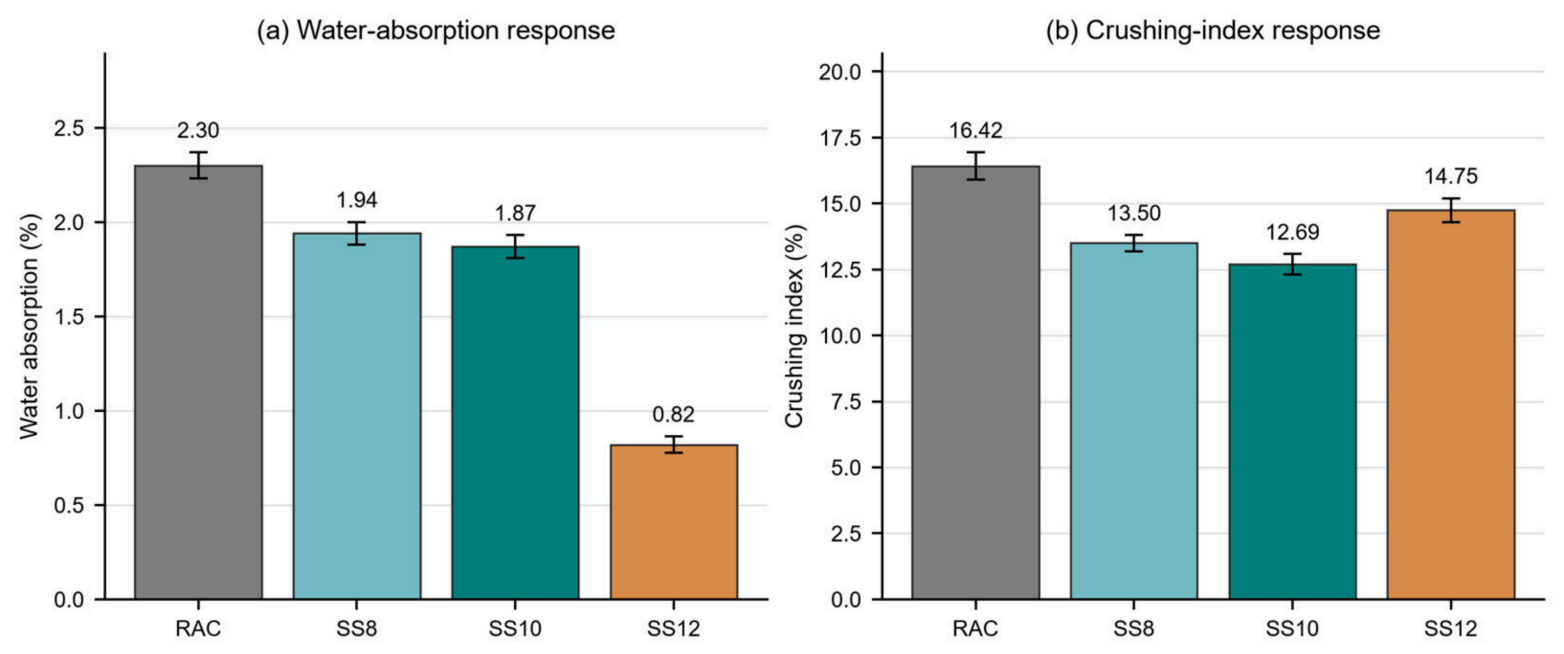
Water absorption and crushing index of untreated and KH560-treated RCA. Error bars represent SD.

Table 4 summarizes the aggregate results shown in Figure 4. Water absorption was 2.30 ± 0.07% for untreated RCA and 0.40 ± 0.03% for NCA. The SS8, SS10, and SS12 values were 1.94 ± 0.06%, 1.87 ± 0.06%, and 0.82 ± 0.04%, respectively. Relative to untreated RCA, these values corresponded to reductions of 15.7%, 18.7%, and 64.3%.

The largest observed absorption difference between adjacent tested concentrations occurred between 10% and 12%, amounting to 1.05 percentage points. The difference between 8% and 10% was 0.07 percentage points. Three concentrations do not establish a threshold, transition, or continuous response function.

The crushing-index means were 16.42 ± 0.52% for untreated RCA, 13.50 ± 0.30% for SS8, 12.69 ± 0.39% for SS10, and 14.75 ± 0.45% for SS12. Relative to untreated RCA, the treated-group reductions were 17.8%, 22.7%, and 10.2%, respectively. SS10 therefore had the lowest crushing index among the treated groups, followed by SS8 and SS12.

COVs ranged from 2.25% to 6.61% across the aggregate measurements.

### 3.2. Concrete Strength and Comparative Screening

**Figure 5:**
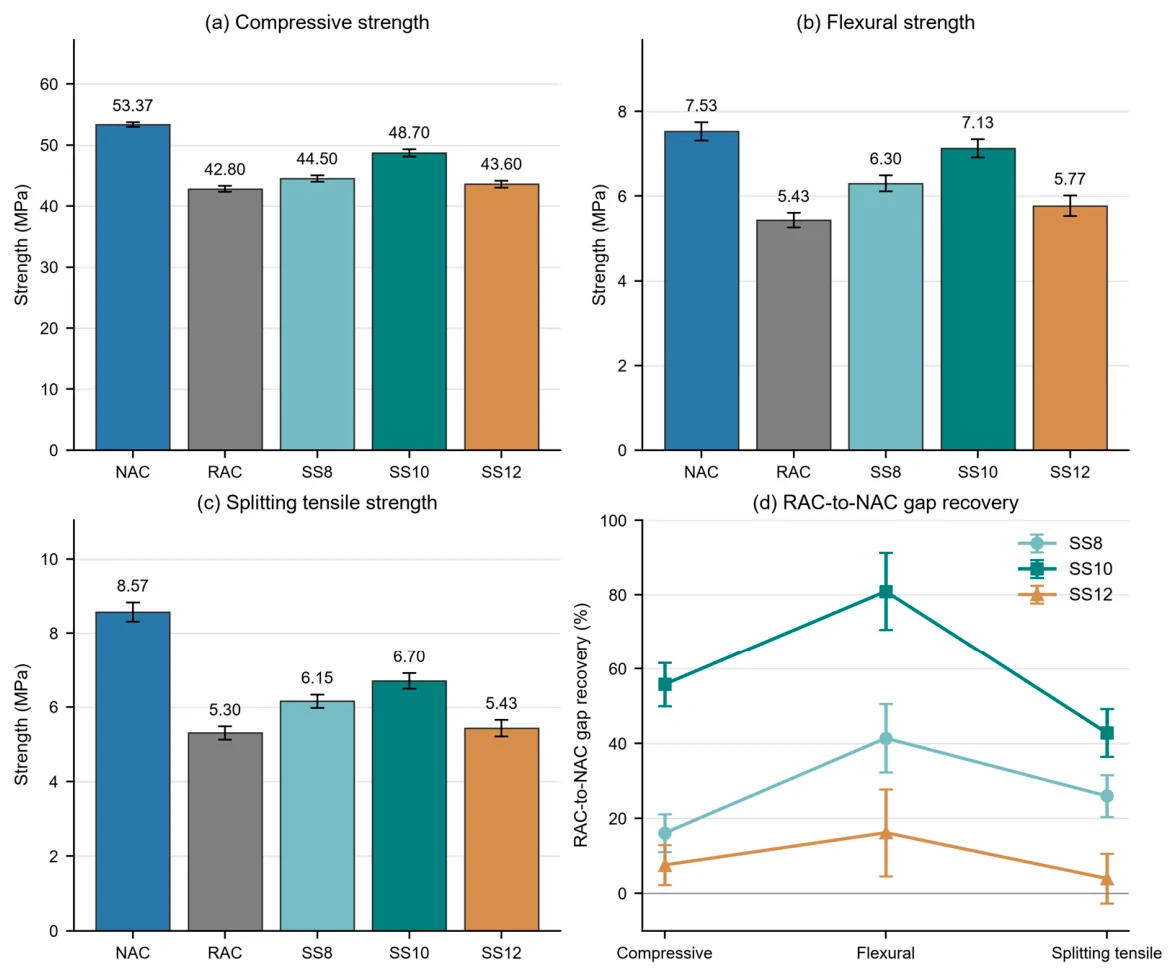
28-day concrete strengths and strength-gap recovery. Error bars show SD in panels (**a**–**c**) and transformed SD in panel (**d**), with reference means fixed.

Table 5 presents the 28-day concrete strength results shown in Figure 5. NAC means were 53.37, 7.53, and 8.57 MPa for compression, flexure, and splitting, respectively. The corresponding RAC means were 42.80, 5.43, and 5.30 MPa.

The exploratory specimen-level ANOVAs gave the following results for compressive strength, *F*(4, 10) = 215.93, flexural strength, *F*(4, 10) = 53.52, and splitting tensile strength, *F*(4, 10) = 117.95 (all *p* < 0.001). Tukey HSD comparisons showed that SS8 exceeded RAC in compression (adjusted *p* = 0.0168), flexure (adjusted *p* = 0.0034), and splitting tension (adjusted *p* = 0.0042). SS10 exceeded RAC for all three properties (all adjusted *p* < 0.001), whereas SS12 did not differ significantly from RAC in compression (adjusted *p* = 0.3846), flexure (adjusted *p* = 0.3397), or splitting tension (adjusted *p* = 0.9381).

SS8-RAC means were 44.50, 6.30, and 6.15 MPa for compression, flexure, and splitting, respectively. They exceeded RAC by 4.0%, 16.0%, and 16.0% and retained 83.4%, 83.7%, and 71.8% of NAC means.

Among the three treated RCA mixtures, SS10-RAC gave the highest mean in each strength test, at 48.70, 7.13, and 6.70 MPa. The corresponding increases over RAC were 13.8%, 31.3%, and 26.4%, with NAC retentions of 91.2%, 94.7%, and 78.2%. Its flexural-strength mean was 0.40 MPa below that of NAC.

SS12-RAC means were 43.60, 5.77, and 5.43 MPa, respectively. These exceeded RAC by 1.9%, 6.3%, and 2.5%, retaining 81.7%, 76.6%, and 63.4% of NAC means. The values were below the corresponding SS8-RAC and SS10-RAC means.

Compressive, flexural, and splitting strength-gap recoveries were 16.1%, 41.4%, and 26.0% for SS8; 55.8%, 81.0%, and 42.8% for SS10; and 7.6%, 16.2%, and 4.0% for SS12. The corresponding equal-weight mean recoveries were 27.8%, 59.9%, and 9.2%. These indices express the same strength results on a normalized scale.

**Figure 6:**
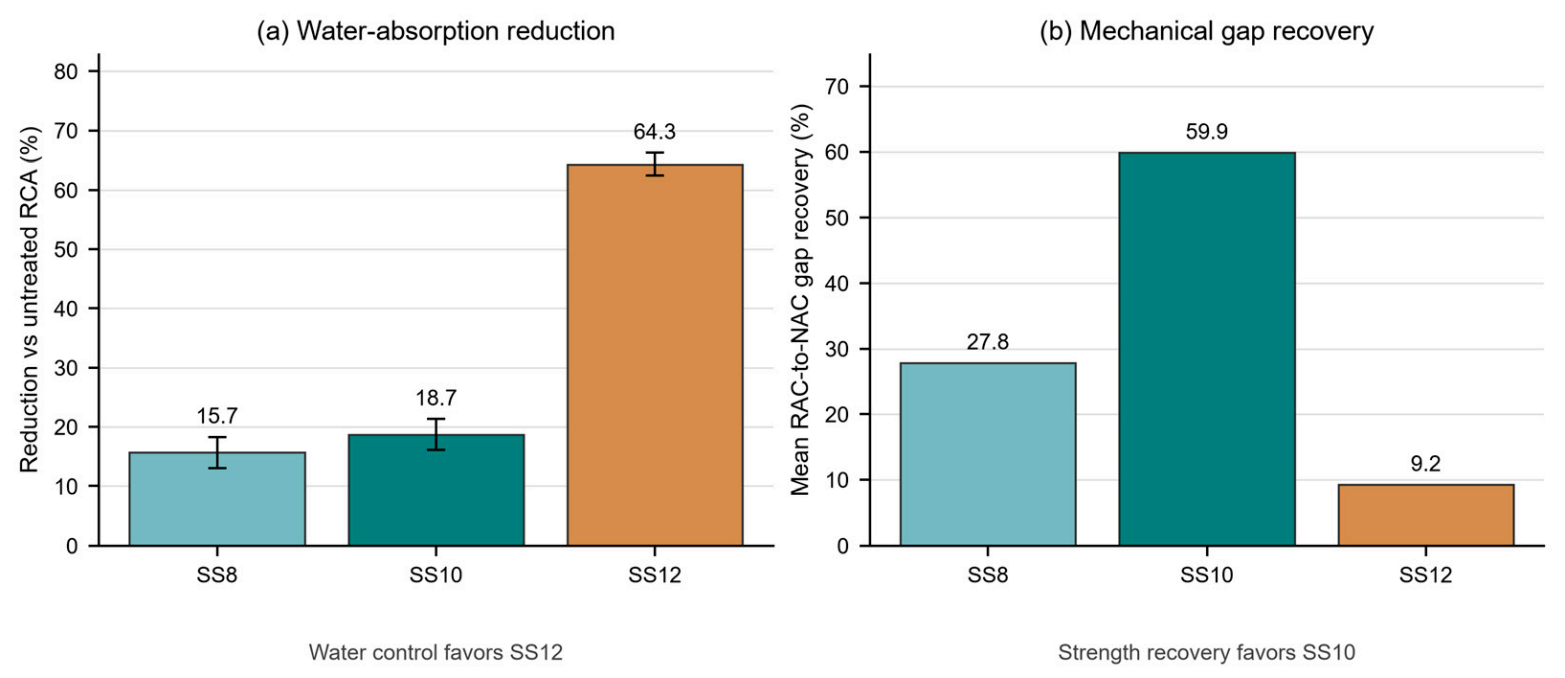
Water-absorption reduction and equal-weight mean strength-gap recovery. Error bars in panel (**a**) show propagated SD with the RCA mean fixed; panel (**b**) shows means only.

**Figure 7:**
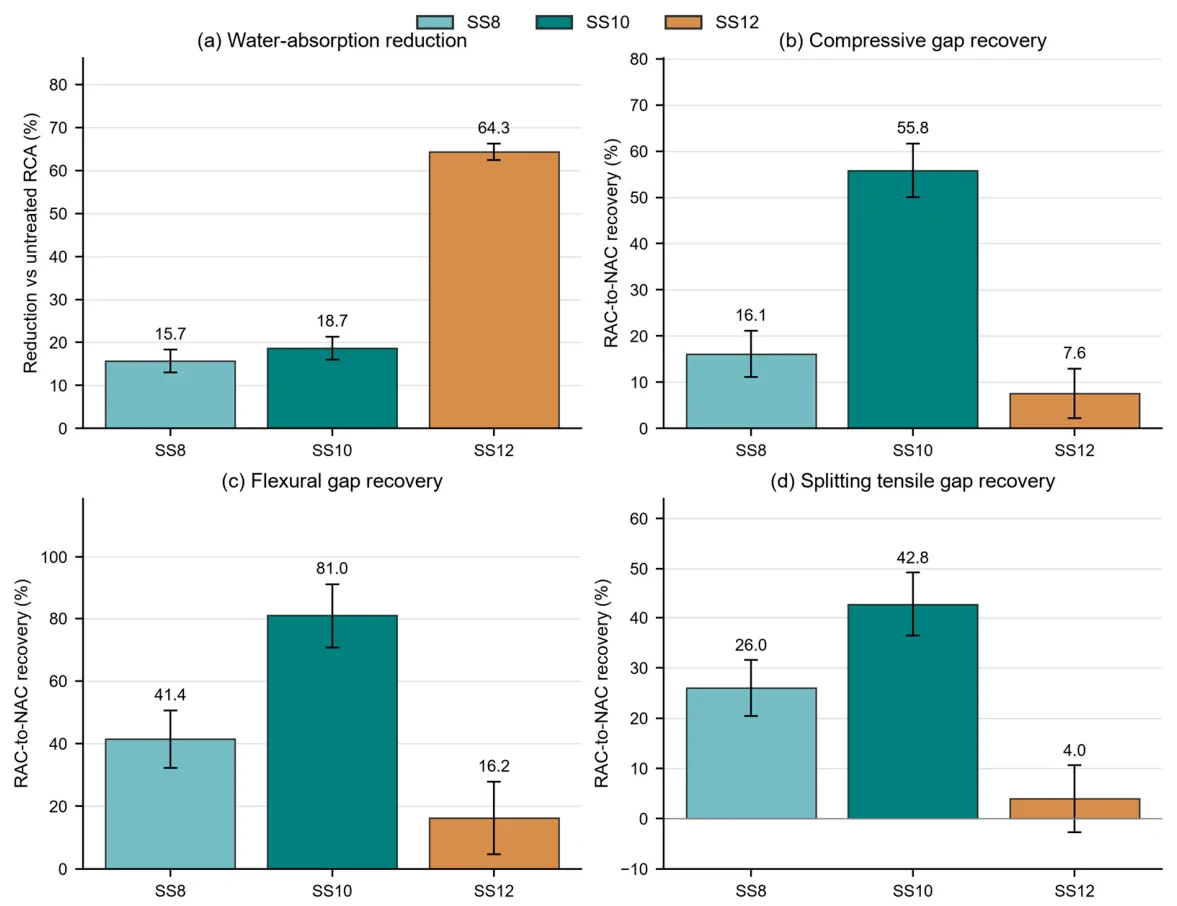
Water-absorption reduction and property-specific strength-gap recovery. Error bars show propagated SD with reference means fixed.

Figure 6 and Figure 7 summarize the screening comparison. At the group-mean level, SS10 dominated SS8 in water-absorption reduction, crushing-index reduction, and all three strength-gap recoveries. SS10 and SS12 remained non-dominated because the absorption and strength rankings differed.

The tested concentrations identify candidate formulations for further comparison rather than a continuous concentration-response relation.

## 4. Discussion

### 4.1. Interpretation of the Aggregate–Concrete Comparison

The absorption and strength rankings did not coincide. SS12 produced the lowest water absorption, whereas SS10 gave the highest means among the three treated RCA mixtures in all three strength tests. The result shows why moisture response and concrete strength should be considered together when screening an RCA treatment.

Several factors could contribute to this pattern, including surface wettability, pore accessibility, aggregate integrity, and paste–aggregate compatibility. These possible mechanisms were not measured directly in this study.

Previous impregnation studies likewise show that transport and mechanical properties can respond differently to surface treatment [19,21]. Those studies provide context for the present group-mean comparison, while their different materials and procedures prevent direct mechanistic confirmation.

The crushing-index pattern in Table 4 suggests that the lowest absorption and the greatest resistance to crushing need not occur at the same treatment concentration. This distinction supports evaluating aggregate water uptake and mechanical response together rather than using absorption alone to rank the treatments.

### 4.2. Mechanistic Interpretation and Literature Comparison

Earlier studies evaluated concrete-level silane application [13], coarse-RCA impregnation at one selected concentration [14], and mixed-aggregate or fiber-containing systems [15,16]. Zhang et al. tested the same 8%, 10%, and 12% range, while also varying treatment duration and RCA replacement [17]. Wang et al. compared EVA treatment with EVA/KH560 co-modification [18]. The present study therefore does not claim the first use of this concentration range. Table 6 compares these designs with the present single-source, full-replacement screening program.

Slurry impregnation for paving concrete [28] and silane treatment of recycled fine aggregate [29] address related applications using different materials and processing routes. Other impregnation studies and reviews also describe treatment-dependent mechanical and transport responses [30,31,32]. These comparisons support evaluating both classes of property, while their different treatment conditions preclude direct transfer of an optimum concentration.

Silane-emulsion studies associate lower sorptivity with hydrophobized capillary surfaces [33], while recent silane-modified recycled-fine-aggregate mortar work demonstrates the value of assessing strength and water-ingress resistance together [34]. These studies provide mechanistic and design context rather than direct confirmation.

One possible explanation is that reduced wettability or pore accessibility limited water entry without producing a proportional improvement in interfacial load transfer. Surface coverage, silane distribution, and paste–aggregate compatibility may also have contributed. These mechanisms were not measured directly, and neither X-ray diffraction (XRD) nor scanning electron microscopy (SEM) characterization was performed.

Contact-angle and Fourier-transform infrared spectroscopy (FTIR) measurements could test wettability and surface chemistry, while SEM coupled with energy-dispersive X-ray spectroscopy (EDS) could examine local morphology. XRD and pore-structure measurements would provide complementary evidence.

**Table 6:** Comparison with silane-based recycled-aggregate studies.

Study	Material and Treatment	Evidence Emphasized	Difference from Present Design
Zhu et al. [13]	RAC; surface or integral silane water repellent	Strength and transport-related durability	Concrete-level treatment; no coarse-RCA concentration screen
Bao et al. [14]	0%, 30%, and 50% coarse RCA; 8 wt% silane impregnation	Absorption, chloride transport and SEM	Single dose and partial replacement; durability focus
Huang et al. [15]	Mixed brick/RCA concrete; 10% silane-treated brick aggregate	Compression and microstructural observations	Varied brick/RCA ratio, not silane concentration; different source
Muthuraman et al. [29]	Reactive powder concrete with silane-treated recycled fine aggregate	Mechanical response and durability	Fine aggregate and RPC matrix; no coarse-RCA concentration screen
Zhang et al. [17]	Waste-concrete RCA; 8%, 10%, 12% KH-560; 30-90 min; 20-60% RCA	Water absorption, slump, 28-day compressive strength, and SEM	Orthogonal multi-factor design; concentration not isolated; partial replacement and one strength mode
Wang et al. [18]	Recycled concrete; EVA alone versus EVA/KH560 co-modification	Compression, shear, SEM/XRD/FTIR, and molecular dynamics	Polymer-assisted treatment; no standalone coarse-RCA KH560 concentration screen
Present study	Single airport coarse-RCA source; nominal 8%, 10%, 12% solutions; 100% replacement	Aggregate properties with SD/COV and three 28-day strengths with exploratory specimen-level analysis	Single-source screening; common target grading and nominal effective w/c; one concrete batch per mixture

### 4.3. Limitations and Requirements for Pavement-Specific Validation

The findings provide a laboratory comparison for one airport-pavement RCA source. Table 7 summarizes the treatment priorities identified from the measured aggregate properties and 28-day strength means.

Parent-concrete age, original strength grade, carbonation depth, and chloride condition were not documented. The results are therefore interpreted for the investigated RCA source.

Drying shrinkage was not measured; the present screening is therefore limited to aggregate properties and 28-day concrete strengths. Because an ethanol–water carrier-only control was not included, the contribution of the carrier treatment cannot be separated from that of KH560 in comparisons with untreated RAC.

The lowest water absorption and highest strength means occurred at different tested concentrations. This trade-off supports selecting candidate formulations for further evaluation under the intended pavement service conditions.

## 5. Conclusions

1. One airport-pavement RCA source was tested at nominal KH560 treatment-solution concentrations of 8%, 10%, and 12% with 100% coarse-aggregate replacement. Aggregate properties and 28-day concrete strength means were compared under a common target grading and nominal effective water-to-cement ratio of 0.360.

2. Relative to untreated RCA, SS8, SS10, and SS12 showed crushing-index reductions of 17.8%, 22.7%, and 10.2%, respectively. SS10 had the lowest crushing index among the treated groups, whereas SS12 showed the largest water-absorption reduction, at 64.3%.

3. Among the three treated RCA mixtures, SS10 gave the highest mean compressive, flexural, and splitting tensile strengths, exceeding the RAC means by 13.8%, 31.3%, and 26.4%. Its flexural-strength mean reached 94.7% of the NAC mean.

4. Water absorption and concrete strength favored different nominal treatment-solution concentrations. SS10 is therefore the stronger candidate for further strength-oriented testing, whereas SS12 is the stronger candidate when water-absorption reduction is the primary screening criterion. The three concentrations do not define a universal optimum or concentration threshold.

## Figures and Tables

**Table 1:** Baseline properties of natural coarse aggregate and wet-processed RCA.

Aggregate	Water Absorption (%)	Moisture (%)	Apparent Density (kg m^−3^)	Fines (%)	Flaky/ Elongated (%)	Crushing Index (%)
NCA	0.40	0.30	2701.49	0.35	3.52	8.50
Wet-processed RCA	2.30	1.62	2611.92	0.62	4.52	16.42

**Table 2:** Research program: concrete mixture proportions (kg m^−3^ unless otherwise indicated).

Mixture	Cement	Sand	NCA	RCA	Effective Water.	Water Reducer	Additional Water	Effective w/c
NAC	570	613	1012	0	205	2.85	0	0.360
RAC	570	613	0	1012	205	2.85	19.23	0.360
SS8-RAC	570	613	0	1012	205	2.85	15.58	0.360
SS10-RAC	570	613	0	1012	205	2.85	14.88	0.360
SS12-RAC	570	613	0	1012	205	2.85	4.25	0.360

**Table 3:** Test scope and available specimen records.

Test	Groups Represented	Sample/Specimen Record	Age	Method Reference
Aggregate absorption	NCA, RCA, SS8, SS10, SS12	3 independent aggregate samples per group	Not applicable	GB/T 14685-2022 [26]
Crushing index	NCA, RCA, SS8, SS10, SS12	3 independent aggregate samples per group	Not applicable	GB/T 14685-2022 [26]
Compression	All five mixtures	100 mm × 100 mm × 100 mm; 3 per mixture	28 d	GB/T 50081-2019 [27]; see Section 2.4
Flexure	All five mixtures	100 mm × 100 mm × 400 mm; 3 per mixture	28 d	GB/T 50081-2019 [27]; see Section 2.4
Splitting tension	All five mixtures	100 mm × 100 mm × 100 mm; 3 per mixture	28 d	GB/T 50081-2019 [27]; see Section 2.4

**Table 4:** Aggregate properties and changes relative to untreated RCA.

	Water Absorption (%)			Crushing Index (%)		
Material	Mean ± SD	COV (%)	Reduction vs. RCA (%)	Mean ± SD	COV (%)	Reduction vs. RCA (%)
NCA	0.40 ± 0.03	6.61	—	8.50 ± 0.30	3.58	—
Untreated RCA	2.30 ± 0.07	3.04	0.0	16.42 ± 0.52	3.18	0.0
SS8	1.94 ± 0.06	3.14	15.7	13.50 ± 0.30	2.25	17.8
SS10	1.87 ± 0.06	3.25	18.7	12.69 ± 0.39	3.09	22.7
SS12	0.82 ± 0.04	5.32	64.3	14.75 ± 0.45	3.07	10.2

SD, standard deviation; COV, coefficient of variation.

**Table 5:** 28-day concrete strengths and Tukey HSD groupings.

	28-Day Strength (MPa)			
Mixture	Compressive	Flexural	Splitting Tensile	n
NAC	53.37 ± 0.37 ^a^ (COV 0.70%)	7.53 ± 0.22 ^a^ (COV 2.89%)	8.57 ± 0.26 ^a^ (COV 3.05%)	3
RAC	42.80 ± 0.49 ^d^ (COV 1.15%)	5.43 ± 0.17 ^c^ (COV 3.21%)	5.30 ± 0.17 ^c^ (COV 3.29%)	3
SS8-RAC	44.50 ± 0.53 ^c^ (COV 1.19%)	6.30 ± 0.19 ^b^ (COV 3.04%)	6.15 ± 0.18 ^b^ (COV 2.97%)	3
SS10-RAC	48.70 ± 0.61 ^b^ (COV 1.26%)	7.13 ± 0.22 ^a^ (COV 3.04%)	6.70 ± 0.21 ^b^ (COV 3.12%)	3
SS12-RAC	43.60 ± 0.56 ^cd^ (COV 1.29%)	5.77 ± 0.24 ^bc^ (COV 4.22%)	5.43 ± 0.22 ^c^ (COV 4.00%)	3

Values are mean ± SD; COV is shown in parentheses. Within each strength column, superscript letters a–d denote the exploratory Tukey HSD groupings (α = 0.05). Means sharing at least one letter do not differ significantly; means with no letter in common differ significantly.

**Table 7:** Candidate priorities for further laboratory screening.

Candidate	Observed Absorption Priority	Observed Strength Priority	Interpretation
SS10	Absorption below untreated RCA	Highest strength means among the three treated RCA mixtures	Candidate for further strength-oriented screening
SS12	Lowest recorded absorption	Lower strength means than SS10	Candidate for further absorption-oriented screening

## Data Availability

Summary results are included in the article. The individual concrete-specimen results and aggregate replicate measurements are available from the corresponding author upon reasonable request.

## Abbreviations

RCA, recycled coarse aggregate; NCA, natural coarse aggregate; RAC, concrete containing untreated RCA; NAC, natural-aggregate concrete; KH560, 3-glycidoxypropyltrimethoxysilane; SS8, SS10, and SS12, RCA treated at nominal KH560 treatment-solution concentrations of 8%, 10%, and 12%; ITZ, interfacial transition zone; XRD, X-ray diffraction; FTIR, Fourier-transform infrared spectroscopy; SEM/EDS, scanning electron microscopy with energy-dispersive X-ray spectroscopy.
